# CGTS: a site-clustering graph based tagSNP selection algorithm in genotype data

**DOI:** 10.1186/1471-2105-10-S1-S71

**Published:** 2009-01-30

**Authors:** Jun Wang, Mao-zu Guo, Chun-yu Wang

**Affiliations:** 1 Department of Computer Science and Engineering, Harbin Institute of Technology, Harbin 150001, PR China

## Abstract

**Background:**

Recent studies have shown genetic variation is the basis of the genome-wide disease association research. However, due to the high cost on genotyping large number of single nucleotide polymorphisms (SNPs), it is essential to choose a small subset of informative SNPs (tagSNPs), which are able to capture most variation in a population, to represent the rest SNPs. Several methods have been proposed to find the minimum set of tagSNPs, but most of them still have some disadvantages such as information loss and block-partition limit.

**Results:**

This paper proposes a new hybrid method named CGTS which combines the ideas of the clustering and the graph algorithms to select tagSNPs on genotype data. This method aims to maximize the number of the discarding nontagSNPs in the given set. CGTS integrates the information of the LD association and the genotype diversity using the site graphs, discards redundant SNPs using the algorithm based on these graph structures. The clustering algorithm is used to reduce the running time of CGTS. The efficiency of the algorithm and quality of solutions are evaluated on biological data and the comparisons with three popular selecting methods are shown in the paper.

**Conclusion:**

Our theoretical analysis and experimental results show that our algorithm CGTS is not only more efficient than other methods but also can be get higher accuracy in tagSNP selection.

## Background

Recent studies show that the abundance of single nucleotide polymorphisms (SNPs) and haplotypes can provide the most complete information for genome-wide association studies. Through the analysis of SNPs and haplotypes, most of the genetic variations among different people can be discovered. However, due to the excessive SNPs, which are about 10 million in the human genome [1-3], it is costly to genotyping and studying all SNPs in a candidate region for a large number of individuals. Thus the SNP selecting strategy is proposed to find only a subset of SNPs, which are called tagSNPs or tagging SNPs, to represent the whole SNP set. These tagSNPs have high linkage disequilibrium (LD) values with the rest SNPs [4], and the genetic variation information they have are enough to support the further study, such like disease association gene identification and population variation finding [5,6].

Several computational methods have been proposed to solve the tagSNP selecting problem. One common approach is based on the haplotype blocks partitions. These methods delimit the human genome into a set of discrete blocks, where only a small set of common haplotypes exist. These methods search the minimum subsets of tagSNPs from each block. The selected tagSNPs can distinguish each pair of common haplotypes in the block [7], or at least most of them [8-11]. However, there is no general solution on how the blocks are formed. And the lack of the inter-block association degrades the selection accuracy. The LD based methods use the pairwise associations of SNPs. TagSNPs are selected from the site clusters which consist of SNPs with strong LD association (measured by the pairwise LD value *r*^2^) between each other [12-15]. These tagSNPs can represent associated SNPs in long distance without the block restriction, but may lose some important information contained in the rest SNPs and fail to distinguish all haplotypes in a LD cluster. Bafna. et al.[16] proposed a somewhat different approach, which assumes tagSNPs can reconstruct the remaining SNPs of an unknown sample with high accuracy. TagSNPs are selected by the measure called *informativeness*, which quantifies how well the unselected SNPs are predicted and the complete haplotypes are reconstructed through the selected SNPs [17-19]. These methods do not need prior block partitioning or limited haplotype diversity, but their performances are limited by the restrictions such as the fixed number of tagSNPs and the definitions of the *informativeness*.

In this paper, we present a new method based on clustering and graph, which is called CGTS, to select tagSNPs. Unlike the previous methods, our method integrates the information of the LD association and the genotype diversity using the site graphs without the information loss and the limit of block partition. Graph based algorithm uses the genotype information to discard the redundant SNPs, and does not need to define block or fix on the tagSNP number. To avoid high computational complexity of graph algorithm for large data, the clustering algorithm is proposed to process the genotype data. Compared to three existing popular methods, our method can give better performance in the experiments.

## Results and discussion

### Implementation and data

We implemented our clustering and graph based tagSNP selection algorithm (CGTS) in C ++ and run the program on a PC with a 1.4 GHz CPU and 512 MB memory. A genotype phasing algorithm: PHASE [20] is used on the genotype data to obtain the haplotypes for other methods such as HapBlock and STAMPA compared in experiments.

Five public biological data were used for evaluation. These data include: the Hapmap data set of human chromosome 21 which has 20163 SNPs for 90 European persons [21]; the IBD 5q31 data set which have genotypes for 103 SNPs [8] from an inflammatory bowel disease study of father-mother-child trios, and we only used the children's data; three encode regions ENm013, ENr112 and ENr113 from HapMap [21], The number of SNPs genotyped in each region is 361, 412 and 515. All missing and ambiguous alleles are deleted from the test data.

### Evaluation method

The evaluation method is based on the accuracy of non-tagging SNP prediction by the tagSNPs. The prediction accuracy is determined by cross-validation [22]. The data set is divided into ten subsets. The algorithm is run on the nine subsets to select a minimum set of tagSNPs. The nine subsets and the tagSNPs in the remaining set are used to predict the non-tagging SNPs in the remaining set.

The prediction of non-tagging SNPs is based on the assumption that given the genotype values of two SNPs, the probabilities of the values at any intermediate SNPs do not change by knowing the values of additional distal ones [18]. It means that for each non-tagging SNP *p*, the value of *p *in given genotype sequence can be identified by the value of two closest tagSNPs in the same sequence. Formally, this assumption can be stated as:

(1) $$\begin{array}{l} \forall p:a<p<b,\forall q:q<a\text{ or }q>b,\forall v\in \{0,1,2\},\forall i:\\ \;\mathrm{Pr}[g_{i,p}=v|g_{i,a},g_{i,b}]\approx \mathrm{Pr}[g_{i,p}=v|g_{i,a},g_{i,b},g_{i,q}]\; \end{array}$$

*For an unidentified p*:

(2) $$g_{i,p}=\underset{v\in \{0,1,2\}}{\arg\max}\mathrm{Pr}[g_{i,p}=v|g_{i,a},g_{i,b}]$$

where *a, b *are the two closest tagSNPs of *p*. *g*_*i*, *x *_is the allele value of SNP *x *in genotype *i. q *is the tagSNP which is different from *a, b*.

The prediction precision of one subset is calculated by following equation:

(3) *P*_*i *_= *N*_*c*_*/N*_*a *_

*N*_*c *_is the number of correctly predicted alleles and *N*_*a *_is the number of all predicted alleles.

The accuracy of the algorithm can be computed as:

(4) $$P=\sum_{i=1}^{d}P_{i}/d$$

### Experimental results for trade-off test

In this subsection, we discuss the trade-off between efficiency and solutions of CGTS. CGTS uses a K nearest neighbour (KNN) clustering algorithm to partition the large SNP set and improve the efficiency of tagSNP selection. Different values of clustering size *k *result in different accuracy of the tagSNP selection. By increasing *k*, the solutions of CGTS can be improved but the efficiency may be sacrificed. The efficiency of CGTS is measured by the running time of CGTS with different *k*. The solution improved by different *k *is measured by the improved ratio of algorithm. The improved ratio is calculated by *(N*_50_-*N*_*k*_*)/N*_50_*100%, where *N*_*k *_is the number of tagSNPs found by CGTS with clustering size *k *and 50 is the minimum value of *k *in CGTS. When *k *< 50, the information of SNP cluster is insufficient to find real tagSNPs.

The trade-off tests use the data of human chromosome 21. The 20163 SNPs are firstly divided into four subsets according to their physical distances. Each subset contains 5000 SNPs (the last has 5163 SNPs). CGTS run on four subsets separately and the final tagSNPs are the union of all results on these subsets.

**Figure 1:**
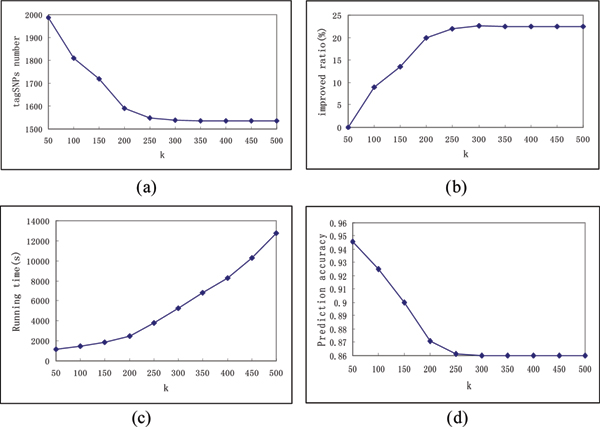
Performances of CGTS on different *k*.

Figure 1a, 1b plot the selected tagSNP number and the improved ratio of tagSNP number with respect to *k*. The *x*-axis stands for *k *and the *y*-axis stands for the selected tagSNP number and the improved ratio of CGTS on chromosome 21 dataset. The change of running time with increasing *k *is plotted on figure 1c. The *x*-axis stands for *k *and the *y*-axis stands for the total running time of CGTS on chromosome 21 dataset. The accuracy decrease is also plotted in figure 1d. The *x*-axis stands for *k *and the *y*-axis stands for the accuracy of CGTS on chromosome 21 dataset.

It can be observed that the improved ratio grows rapidly following the increase of *k *until *k *= 200, after that a more slow improvement is observed. It can be explained by the reduced LD association between SNPs and the stability of information contained by SNP cluster. The elapsed time also increases as *k *becomes large. It is because the sites in the graph increase and more sites need to be investigated with the selecting algorithm to obtain the tagSNPs. On the other hand, the accuracy of CGTS decreases as *k *becomes large. One intuitive reason is that the CGTS discards some real tagSNPs in the selecting step. Following the increase of *k*, the graphs in selecting step become larger and noisy sites also become more in the graph. These noisy sites decrease the LD associations in the graphs and the genotype diversity information they have may interfere with the tagSNP selection. Some real tagSNPs are discarded due to this interference and pseudo-tagSNPs may be put into the result.

As a result, the parameter *k *of CGTS is best set to around 200 to obtain the best improvement in solutions, which still keeps the running time and accuracy within a reasonable period.

### Experimental results on biological data

We compare our algorithm with three recent algorithms for tagSNP selection that are widely used: HapBlock [9], MLR-tagging [23], STAMPA [18] These three softwares represent three common methods for tagSNP selection. HapBlock bases on the block-partition, uses dynamic programming and EM subroutine to get the tagSNPs for each blocks. MLR-tagging uses a multiple linear regression approach and selects tagSNPs based on LD associations. STAMPA uses the SNP prediction accuracy for other SNPs to select tagSNPs. HapBlock and the training step of STAMPA use the haplotype data as input. MLR-tagging and STAMPA need to fix on the tagSNP number. Therefore, to compare the performance of our method to these methods, the tagSNP number of MLR and STAMPA are same as the tagSNP number obtained by our algorithm.

**Figure 2:**
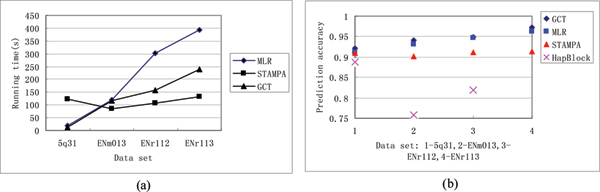
Performances of four selection methods.

The comparison results of the three methods on the ENm013, ENr112, ENr113 and 5q31 are shown in Table 1 and figure 2. HapBlock gave no solution for ENr113 due to the memory overload.

**Table 1:** The comparison of the four methods

	Datasets	Hap-Block	MLR	STAMPA	CGTS
Number of tagSNPs	5q31	17	8	8	8
	
	ENm013	15	12	12	12
	
	ENr112	33	20	20	20
	
	ENr113	~	22	22	22

Prediction accuracy	5q31	0.887	0.912	0.909	0.922
	
	ENm013	0.757	0.932	0.901	0.941
	
	ENr112	0.819	0.947	0.911	0.949
	
	ENr113	~	0.963	0.913	0.972

Run time(s)	5q31	19100	18	122	14
	
	ENm013	9207	121	85	117
	
	ENr112	4405	303	108	156
	
	ENr113	~	394	133	239

CGTS can perform better than HapBlock in prediction accuracy, get smaller tagSNP sets and use shorter times. It is because that there is no need to conduct block partition and the LD associations among SNPs are taken into consideration. CGTS is more accurate than MLR-tagging and STAMPA with the same number of selected tagSNPs. When the SNP size of the input data is increasing, STAMPA is asymptotically faster but CGTS is more accurate with an acceptable running time. It is due to the increasing iteration number of CGTS. Appropriate choosing of *k *can reduce the running time of CGTS. And we chose the *k *= 250 because the CGTS can obtain better improvement in solutions but still keep the running time and accuracy within a reasonable period on the given biological data.

## Conclusion

We investigate a novel hybrid method for SNP-tagging, which is called CGTS. The efficiency and solutions of SNP-tagging are improved by the combination of graph algorithm and site clustering in CGTS. As shown in the experimental results, our algorithm is able to get higher prediction accuracy than other approaches with the same size of tagSNPs, and outperforms these approaches in terms of the tagSNP size. Computational time required by CGTS is quite reasonable and can be tailored to available computing resources as needed. The key component of CGTS is the SNP graph model which integrates the information of the LD association and the genotype diversity. This model makes the SNP-tagging problem transform to a graph pruning problem. Using this model can avoid the information loss of SNPs. Our method does no need to fix the tagSNP number. The tagSNPs are not restricted to any bounded location, and can easily be applied to cover the whole chromosome. In the algorithm, multiple tagSNPs are used to represent each untagged SNPs, which further reduce the number of selected tagSNPs. Another advantage of CGTS is that it is amenable to further computational improvements. For example, parallel programming could be used to search tagSNPs in separate precincts, and further speed up the computation. In addition, CGTS can also be used on multi-allelic genotype data and haplotype data.

## Methods

### SNP graph construction

We are given *n *genotype sequences, each genotype consist of *m *SNPs. If we assume there are no missing data in the sequences, the *n *sequences can form a n*m matrix *M *where rows are sequences and columns are SNPs, *M*[*i*, *j*] ∈ {0,1,2}, where 0 and 1 are homozygous types which represent the major and minor alleles, and 2 indicates the heterozygous type. A set *G *of genotype sequences distributes in the genome region of given populations follow a functions *P*(*g*_*i*_). *P*(*g*_*i*_) is the frequency of *g*_*i*_, where *g*_*i *_is a genotype in *G*. Like the haplotype, genotype sequences only have a few common patterns in a given genome region and these patterns can be distinguished by the tagSNPs. According to the observation made by several biological studies [12,14], tagSNPs usually have strong LD associations with other relative SNPs, except some special tagSNPs which have no association with other sites. We can represent each genotype by a vector *Ti *∈ {0, 1, 2}^*t*^, where *t *is the size of tagSNP set in the genotype, and *T*_*i *_belongs to *i*-th genotype. Thus, the tagSNPs at least have two important attributes: (1) *T*_*i *_can identify common patterns of given genotype set, and the frequency of *T*_*i*_: *P*(*T*_*i*_) equals to the related *P*(*g*_*i*_); (2) Each tagSNP has the strong LD value with its relative site set. Our goal is to find a minimum size set *ST *of tagSNPs for given genotype set, and these tagSNPs satisfy the two attributes described above. Formally, for a given set *G *and its matrix *M*, our objective is to find a set of tagSNPs *ST *with minimum size, and for each pair of common genotype pattern *P*_*i *_and *P*_*j *_in the *G*, these is a tagSNP *s*_*i *_∈ *ST *such that *M*[*k*, *i*] ≠ *M*[*k*, *j*]. In addition, the average LD value of *s*_*i *_with its relative sites is highest in the related site set.

To achieve this goal, the main problem is how to represent the two features of tagSNPs and associate them to find the real tagSNPs. For a given genotype set *G*, all SNPs are distributed randomly as discrete sites in the space. If these sites are connected according to the genotype diversity information and the length of edge is set based on the LD value of two sites, the SNPs can form a graph. Through the analysis of several biological data, we discovered that the genetic-relative sites are bunched up in the graph and there are usually more sites around the real tagSNPs than the others except some special tagSNPs which have no association with any sites. That means it is feasible to construct a site graph to describe the SNP attributes and use a graph algorithm on it to find tagSNPs.

For a given genotype set *G*, the SNP set is *S *(|*S*| = *m*), and the LD value matrix is *R*. Each SNP has some information of the genotype diversity. Therefore, for each site *s*_*i*_, we define a *diversity set X*_*i *_= {$X_{i}^{0},X_{i}^{1},X_{i}^{2}$} to represent genotype diversity information for SNP site *s*_*i*_. $X_{i}^{j}$ = {*x*_*r*_*| x*_*r *_= *r*, *r *∈ [1, n]}, for *j *= 0,1,2, n is the size of the input genotype set size, *r *is the sequence number of a genotype in given set, *x*_*r *_∈ $X_{i}^{j}$ means the *r-th *genotype have the value *j *at *i*-*th *SNP. If *X*_*i *_has two Φ subsets, which means the SNP has only one value at all genotypes, we assume this site has no diversity information, and delete it before the graph construction. If two *diversity sets X*_*a *_= *X*_*b*_, we assume these two sites have the same diversity information, and these sites can combine to one site in the graph for their similar information. However, it is too complex to combine the whole SNP *diversity sets *and their LD associations in one graph. Thus, the *X*_*i *_is divided to three subsets $X_{i}^{0},X_{i}^{1},X_{i}^{2}$, and three graphs are constructed for *S*. In each graph, only the information of one subset is represented. The selection results for three graphs are combined to get the final tagSNPs. For a given *S *and the *diversity subset *$X_{i}^{j}$ for each *s*_*i*_, *j *∈ {0, 1, 2}, the site graph *G*_*j*_(*V, E*) can be constructed as following steps:

(1) The site set *V*: each site *v*_*i *_of *V *represents a SNP *s*_*i *_of *S*. Each *v*_*i *_corresponds to the *diversity subset *$X_{i}^{j}$ of *s*_*i*_. Each *v*_*i *_has a site weight *ws*_*i *_defined by the number of the sites similar to *s*_*i*_. For two SNPs *s*_*a *_and *s*_*b*_, *s*_*a *_is similar to *s*_*b *_in graph *G*_*j *_when $X_{a}^{j}=X_{b}^{j}$. *ws*_*i *_is computed from two sets: the sites satisfy *X*_*a *_= *X*_*b *_and the sites satisfy *X*_*a *_≠ *X*_*b *_and $X_{a}^{j}=X_{b}^{j}$.

(2) The edge set *E*: tagSNPs can identify all or at least most of the common genotype patterns. The diversity information is represented by *X*_*i*_. The associated SNPs have their *X*_*i *_overlap with each other. The overlapping of *X*_*i *_causes the redundancy. TagSNPs are selected to delete these redundancies. The directed edges between *v*_*i *_are identified to describe these overlapping relationships. $X_{a}^{j}$ and $X_{b}^{j}$ are *diversity subsets *of sites *a*, *b*. If $X_{a}^{j}⊃X_{b}^{j}$, there is a directed edge *e *from *a *to *b*. If $X_{a}^{j}\subset X_{b}^{j}$, there is a directed edge *e *from *b *to *a*. If $X_{a}^{j}⊄X_{b}^{j}$, $X_{b}^{j}⊄X_{a}^{j}$, $X_{a}^{j}\cap X_{b}^{j}$, there is bi-directed edge *e *between *a *and b. If $X_{a}^{j}\cap X_{b}^{j}$, there is no edge between them.

(3) The edge weight set *W*: for edge *e *between sites *a *and *b*, the edge weight *w*_*ab *_= *R *[*a*, *b*]. *R*[*a*, *b*] is the LD value *r*^2 ^of the SNP *a *and the SNP *b*. The LD value *r*^2 ^is usually chosen to evaluate the correlation between two SNPs [24,25], and can be computed as follow:

(5) $$r^{2}=\sum_{i=0}^{m}\sum_{j=0}^{n}p_{i}p_{j}\left|D_{ij}^{'}\right|$$

(6) $$D_{ij}^{'}=\{\begin{array}{ll} 1 & \text{if }D_{ij}=0\\ \frac{D_{ij}}{D_{\max}} & \text{if }D_{ij}\neq 0 \end{array}$$

(7) *D*_*ij *_= *x*_*ij *_- *p*_*i*_*p*_*j*_

(8) $$D_{\max}=\{\begin{array}{ll} \min\text{[}p_{i}(1-p_{j}),p_{j}(1-p_{i})\text{]} & \text{if}D_{ij}>0\\ \min\text{[}p_{i}p_{j},(1-p_{i})(1-p_{j})\text{]} & \text{if}D_{ij}<0 \end{array}\;$$

**Figure 3:**
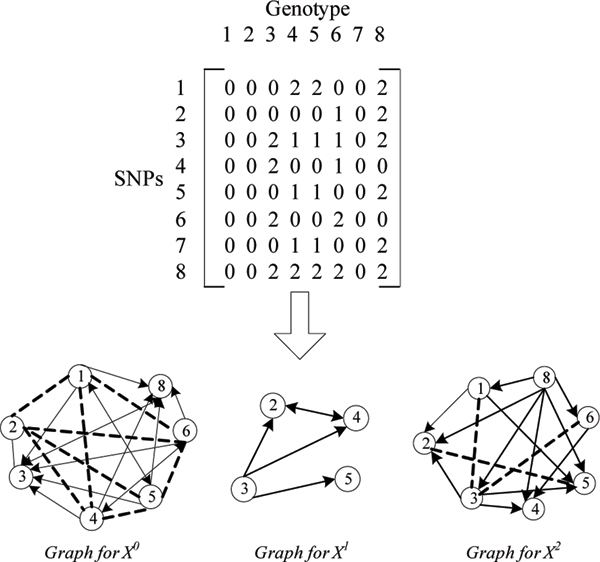
**An example of SNP graphs**. The dashed represents the bi-directed edge in the graph.

In these equations, *p*_*i *_denotes the frequency of *i-th *allele for SNP *a*, *p*_*j *_denotes the frequency of *j-th *allele for SNP *b. m *= *n *= 1, when the data are bi-allele haplotypes; *m *= *n *= 2 for the bi-allele genotype data. *x*_*ij *_is the frequency of observing *i-th *allele for SNP *a *and *j-th *allele for SNP *b *together in the same genotype (haplotype) sequence.*D*_*max *_is the maximum value of LD between the *i-th *allele for SNP *a *and *j-th *allele for SNP *b*. A *r*^2 ^value of 1 indicates the highest LD while 0 indicates no LD. The site graphs of a simple genotype data are shown in figure 3.

### TagSNP selection

In this section we present our selection algorithm. The algorithm is based on the observation that the SNPs with same genotype diversity information and high correlation are congregated into a subgraph through the construction of SNP graphs. The SNPs which have high probability to be tagSNPs usually have a subgraph with higher density. In order to reduce the space and time complexity, we are interested in discarding the redundant sites instead of finding the tagSNPs. Therefore, for each site graph *G*_*j*_(*V, E*), the site which has the subgraph of the maximum density and the highest correlation is the top-priority site for tagSNP selection algorithm. For a given SNP set *S *and its site graph *G*_*j*_(*V, E*) for *diversity subset *$X_{i}^{j}$, the algorithm details are described as follow:

(1) Subgraph finding: in the graph *G*_*j*_, a set of subgraphs *N*(*v*_*i*_) is defined to represent a neighbourhood for each site *v*_*i*_. It is induced by every vertex which has a directed edge from *v*_*i *_to it or has an bi-directed edge between *v*_*i *_and it, including *v*_*i *_itself. In order to find the top-priority site and the relative subgraph, the measures for subgraph density and site correlations information are defined as follow:

For each *N*(*v*_*i*_)(*VI, EI*), the subgraph density is:

(9) *λ *= ||*EI*||/||*VI*||

where ||*EI|*| is the edge set size of the *N*(*v*_*i*_) and ||*VI*|| is the vertex set size of the *N*(*v*_*i*_). The information entropy of the subgraph is defined to describe the correlations information of each *N*(*v*_*i*_) as:

(10) $$EP=\gamma \sum_{i=1}^{c}ws_{i}/c+\sum_{i=1}^{d}\sum_{j=i}^{d}w_{ij}/d$$

where *c *is the number of sites in *N(v*_*i*_) which is equal to ||*VI*||, *d *is the edge size of *N(v*_*i*_) which is equal to ||*EI|*|, *γ *is the normalization factor, *ws*_*i *_is the weight for site *v*_*i *_in the subgraph, *w*_*ij *_is the weight of edge e_*ij *_for the site *v*_*i *_and *v*_*j *_in the subgraph. A high value of *EP *means high LD relations among the sites in the subgraph.

The top-priority site measure function is *T*(*λ*, *EP*):

(11) *T*(*λ*, *EP*) = *θλ *+ *EP*

*θ *is the normalization factor, and the top-priority site is the *v*_*i *_which has the maximum *T *value.

**Figure 4:**
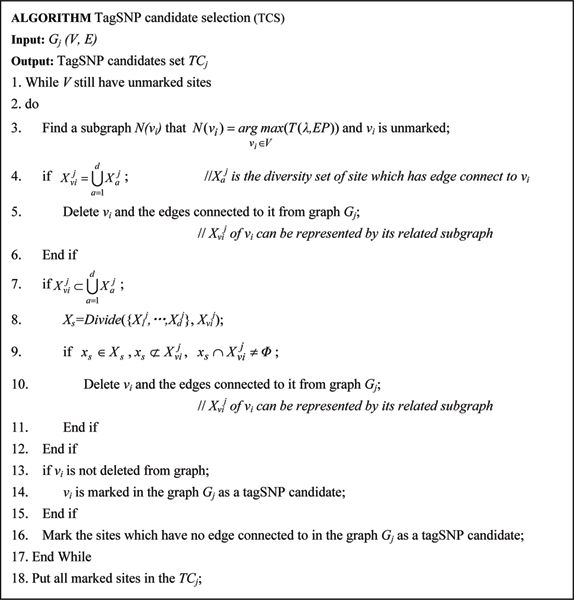
TagSNP candidate selection algorithm.

**Figure 5:**
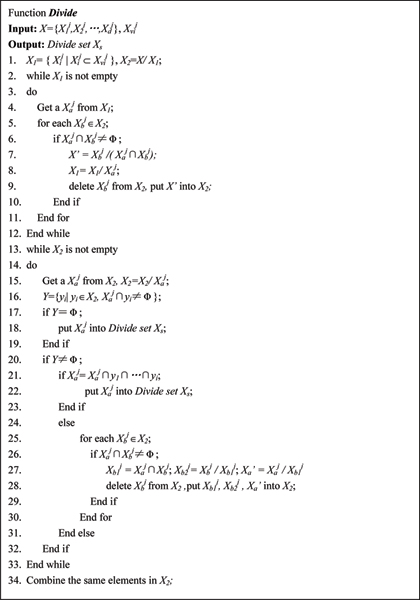
*Divide *function for TagSNP candidate selection algorithm.

(2) TagSNP candidate selection: for each chosen subgraph in the given *G*_*j*_, the tagSNP candidates are selected by evaluating the information overlapping among the sites. A site which is not the candidate is deleted from the graph. The selection algorithm is described in figure 4 and figure 5

(3) Final tagSNP identification: after the tagSNP candidate set *TC*_*j *_for each *G*_*j *_are obtained, the final tagSNPs are identified by a voting mechanism. For a SNP *s*_*i*_, a score function is defined as:

(12) $$\begin{array}{l} score(s_{i},TC_{1},TC_{2},TC_{3})=f(s_{i},TC_{1})+f(s_{i},TC_{2})+f(s_{i},TC_{3})\\ f(s_{i},TC_{i})=\{\begin{array}{ll} 0 & s_{i}\notin TC_{i}\\ 1 & s_{i}\in TC_{i} \end{array} \end{array}$$

**Figure 6:**
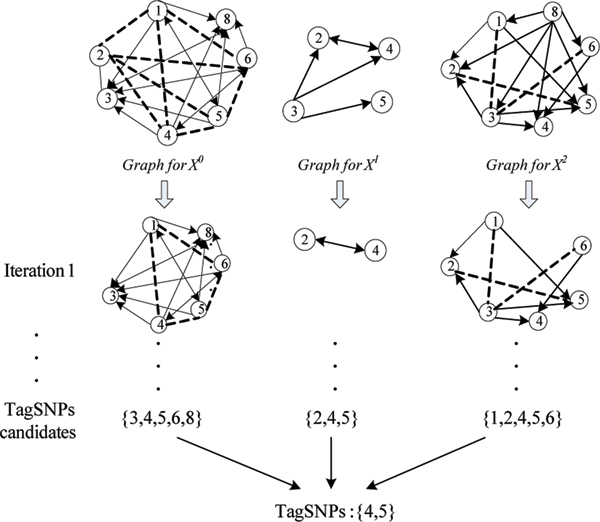
An example of tagSNP selection.

The SNPs which have the highest score are decided to be real tagSNPs. The example of tagSNP selection is given in figure 6. The SNP data are the data used in the SNP graph construction section.

### SNPs clustering

When the size of site graph is larger, the efficiency of our graph algorithm decreases, due to the time-consuming graph construction and tagSNP searching in low-relative SNPs. It is observed that the correlation between SNPs tends to decay as the physical distance increases [8,11,16,26]. That means there are many SNPs have no correlation or less correlation among them and the graph construction and tagSNP searching are unnecessary. Therefore, a clustering algorithm based on KNN is adopted to reduce the complexity of time and space for the input SNP set. The SNP groups which have higher correlation value are selected to construct graph, while the SNPs which have little correlation among them will not appear in the same graph. Due to the correlation decay, the SNP set is firstly divided into several subsets which have SNP number less than 5000. The KNN clustering algorithm is run on these subsets respectively.

In the KNN clustering algorithm, parameter *k *is an integer less than the number of SNPs in the input SNP set *S*, the correlation between two SNPs is represented by the LD value *r*^2 ^calculated through equation (5). In this algorithm, *r*^2 ^is directly related to the distance of two sites. *r*^2 ^= 1, the distance of two SNP sites is 0, and the two SNPs are considered identical and only one of them is retained. *r*^2 ^= 0, the distance of two sites is ∞. *d*_*k*_^*i *^is the LD value of SNP *s*_*i *_and its *k-th *nearest neighbour. In each iteration, the SNP which has the highest *d*_*k*_^*i *^and its *k *nearest neighbours are deleted from *S *and selected to construct the SNP graphs for tagSNP selection. The obtained tagSNPs are put into the *S *and a new iteration is started. The obtained tagSNPs of each subset are combined to get the final set of tagSNPs.

The choice of *k *in the KNN algorithm controls the maximum information of the SNP set and the maximum size of site graphs. In general, different values of *k *result in different accuracy of the tagSNP selection. The bigger *k*, the smaller tagSNPs may be obtained, the more running time and space are cost. In the tagSNP selection, there are two possible ways to select *k*. (1) Select *k *so that the LD value between a chose SNP and its *k*-nearest neighbour is greater some threshold. (2) Select *k *to achieve desired prediction accuracy via cross-validation. The accuracy evaluation is given in more detail in the evaluation section. The trade-off test of *k *is discussed in result section.

### Complexity analysis

For a SNP subset which has the site set size *n *andcontains *m *genotype sequences, the running time for LD value computing is *O*(*m*). Thus, the total running time of the clustering step is *O*(*n*^2^*m*). For each cluster which has (*k*+1) SNPs, constructing site graphs takes *O*(*k*^2^). Finding subgraph needs *O*(*k*). Selecting tagSNP candidates from a subgraph which has *t *sites (0 <*t *≤ *k *+ 1) takes *O*(*t*^2^). Identifying the final tagSNPs needs *O*(*k*). The total running time of tagSNP selection is *O*(*k*^2^+ *kt*^2^+*k*). Since *t *≤ *k*+1, the total running time for whole algorithm is *O*(*n*^2^*m *+ *k*^3^*n*/*k*) = *O*(*n*(*nm *+ *k*^2^)) with taking into the account of the iteration number. In practice, the site set size *n *usuallyless than 5000 and *k *is controlled in interval [50, 500], the running time is less than expectation.

## Abbreviations

SNPs: single nucleotide polymorphisms; LD: linkage disequilibrium; KNN: K nearest neighbour.

## Competing interests

The authors declare that they have no competing interests.

## Authors' contributions

JW proposed the idea, designed the experiments and wrote the paper under the supervision of MG and YW. All authors read and approved the final manuscript.
